# Translating advocacy into action: exploring oncology healthcare professionals’ awareness and use of the Clinical Oncology Society of Australia position statement on exercise in cancer care

**DOI:** 10.1007/s00520-025-09633-0

**Published:** 2025-06-14

**Authors:** Jack Dalla Via, Francesca Cehic, Carolyn J. Peddle-McIntyre, Christopher R. Andrew, David Mizrahi, Yvonne Zissiadis, Nigel A. Spry, Robert U. Newton, Mary A. Kennedy

**Affiliations:** 1 Nutrition and Health Innovation Research Institute, School of Medical and Health Sciences, Edith Cowan University, Perth, WA Australia; 2 Institute for Physical Activity and Nutrition, School of Exercise and Nutrition Sciences, Deakin University, Geelong, VIC Australia; 3 Adelaide Medical School, University of Adelaide, Adelaide, SA Australia; 4 Exercise Medicine Research Institute, School of Medical and Health Sciences, Edith Cowan University, Joondalup, WA Australia; 5 The Daffodil Centre, The University of Sydney, a joint venture with, Cancer Council NSW , Sydney, NSW Australia; 6 Discipline of Exercise and Sports Science, Faculty of Medicine and Health, The University of Sydney, Sydney, NSW Australia; 7 Radiation Oncology, GenesisCare, Perth, WA Australia; 8 School of Human Movement and Nutrition Sciences, The University of Queensland, St Lucia, QLD Australia

**Keywords:** Cancer survivorship, Exercise, Guidelines, Implementation, Oncology

## Abstract

**Purpose:**

The Clinical Oncology Society of Australia (COSA) position statement on exercise in cancer care encourages all healthcare professionals to discuss, recommend, and refer people with cancer to exercise; however, use of these recommendations in practice is unknown.

**Methods:**

Oncology healthcare professionals working in Australia were invited to complete a cross-sectional online survey that assessed contextual factors that influence implementation of COSA exercise guidance in cancer care, based on the Consolidated Framework for Implementation Research.

**Results:**

We received 133 survey responses. Most were women (74%), nurses (35%), or oncologists (26%), involved in cancer care for > 10 years (63%), and in a public hospital setting (69%). Most participants agreed that exercise is beneficial (94%) and the COSA recommendations would positively influence patients’ exercise behaviours (94%). However, only 35% routinely apply COSA recommendations in practice, and only 35% believe they are the best person to provide exercise support. Patient-level barriers included needing additional support to access exercise (92%), most commonly financial (74%). Organisational-level barriers included a lack of dedicated resources to support delivering exercise guidance (69%), and not believing providing exercise guidance is an important part of their role (51%). Only 24% agreed their organisation revised practice based on the COSA recommendations.

**Conclusion:**

Despite most oncology healthcare professionals agreeing that exercise is beneficial, and that the COSA recommendations are important for patients, only a minority actually apply the recommendations in their practice. Targeted implementation efforts are needed to facilitate use of COSA exercise guidance in clinical practice.

**Supplementary Information:**

The online version contains supplementary material available at 10.1007/s00520-025-09633-0.

## Introduction

There is robust evidence of the benefits of exercise for people living with and beyond cancer demonstrating that appropriately prescribed exercise alleviates several physical and psychological treatment-related side effects [1]. Despite this evidence, fewer than 13% of people in Australia reportedly receive exercise guidance as part of their cancer care [2, 3]. The Clinical Oncology Society of Australia (COSA) advocates for more widespread use of exercise as part of cancer care. A 2018 position statement developed by COSA, and endorsed by 20 other organisations, states that exercise should be a standard part of evidence-based cancer care [4]. Specifically, COSA’s position statement encourages all health professionals involved in the care of people with cancer to (1) discuss the role of exercise in cancer recovery; (2) recommend their patients adhere to the exercise guidelines; and (3) refer their patients to a health professional who specialises in the prescription and delivery of exercise [4].

It is widely understood that changes to clinical practice take many years to enact [5], and the integration of exercise into standard cancer care is no exception [6, 7]. Advocacy and policy efforts, such as establishing position statements from organisational leaders in a field, are recognised as an important component of implementing clinical change because they establish the group’s point of view and recommendations on an issue (i.e. COSA’s stand on exercise as an important treatment in cancer care) and help to facilitate access to specific components of care [8, 9]. The COSA position statement was released over 5 years ago, but its influence on the provision of exercise in clinical cancer care is unknown.

Given the continued focus on the benefit of including exercise as a component of cancer care, it is timely to explore the impact of the COSA statement’s guidance on clinical practice. An in-depth understanding of awareness and use of the statement by the clinician’s whom it targets can guide future advocacy efforts. The people who are meant to use COSA’s guidance in practice can provide important insight to steer targeted implementation efforts. Therefore, the purpose of this study was to (1) clarify oncology healthcare professionals’ awareness and practice regarding the COSA position statement for exercise in cancer care, and (2) identify implementation barriers and facilitators to action. The results of this survey will help to develop implementation strategies to underpin the widespread adoption of exercise into routine clinical practice.

## Methods

### Study design and eligibility

A cross-sectional online survey was used to collect data. Eligible participants included any healthcare professional involved in the care of people with cancer in Australia, except for accredited exercise physiologists and physiotherapists as the COSA position statement specifies these professionals as most appropriate to receive referrals of people with cancer. Our survey is reported in compliance with the STROBE guidelines [10]. Ethical approval was obtained from the Edith Cowan University Human Research Ethics Committee (2022–03464-DALLAVIA).

### Survey design

An anonymous English language survey was formulated using Qualtrics (Qualtrics, Provo, UT, USA). The survey was designed using the original Consolidated Framework for Implementation Research (CFIR) [11]. This practical framework was chosen because it is designed to guide the systematic assessment of implementation barriers and facilitators and is widely used within healthcare settings, which will promote the generalisability of the findings. The CFIR framework consists of five domains: intervention characteristics, outer setting, inner setting, characteristics of individuals, and process. Each domain represents a unique element of the implementation context. The research team used the CFIR guide to design the survey questions. Common issues regarding implementation of exercise oncology [6] were queried according to the relevant CFIR domain. No survey questions were included within the process domain as constructs in this domain were outside the scope of the survey aims. Two key steps were taken to increase face validity of the survey. First, an implementation scientist outside of the research team reviewed the survey to verify the interpretation of CFIR constructs. Second, a team of approximately 10 multi-disciplinary clinicians representative of the target population (e.g. nurse manager, medical specialist physicians, pharmacist) pilot tested the survey. Minor amendments to improve the order and wording of questions to improve clarity were made during this two-step process.

Participants could download the Participant Information Letter and provide written informed consent via the survey link. Those who consented were asked screening questions to confirm they were (1) health professionals involved in the care of people with cancer, (2) not accredited exercise physiologists or physiotherapists, and (3) practicing in Australia.

Eligible participants were then taken to the main survey, which contained six sections totalling 36 questions. Section 1 consisted of 12 background demographic questions. Section 2 was comprised of three questions about participant awareness and practice regarding the COSA position statement. Sections 3–6 assessed issues that may influence implementation of the position statement based on relevant constructs within four domains of the 2009 CFIR [11]: (1) intervention characteristics (aspects of the intervention, e.g. evidence-base), (2) outer setting (issues external to the organisation, e.g. patient needs), (3) inner setting (aspects of the healthcare organisation, e.g. culture), and (4) individual characteristics (characteristic of people within an organisation, e.g. knowledge and beliefs). Participants were asked their level of agreement with four statements regarding the role of exercise in cancer care (intervention characteristics). Certain questions were displayed differently depending on participants’ responses. For example, participants who agreed it is easy to discuss/recommend exercise were asked what makes it possible, whereas those who disagreed were asked what makes it difficult. Three questions assessed support needed to access exercise (outer setting). Only participants who agreed that their patients needed support to access exercise were asked what type of support was most needed. Organisational support (inner setting) received was determined by level of agreement with 11 statements. All questions asking for level of agreement used a 5-point Likert scale, ranging from strongly agree to strongly disagree. Participants were then asked if they personally adhered to the National Physical Activity Guidelines (individual characteristics). To try and ensure complete data, participants were not able to skip questions. Finally, participants could enter free text with additional qualitative information in response to the question “Is there anything else you would like to share with about your experience with or feelings about providing exercise discussions, recommendations, and referrals to your patients?”.

### Recruitment

**Fig. 1:**
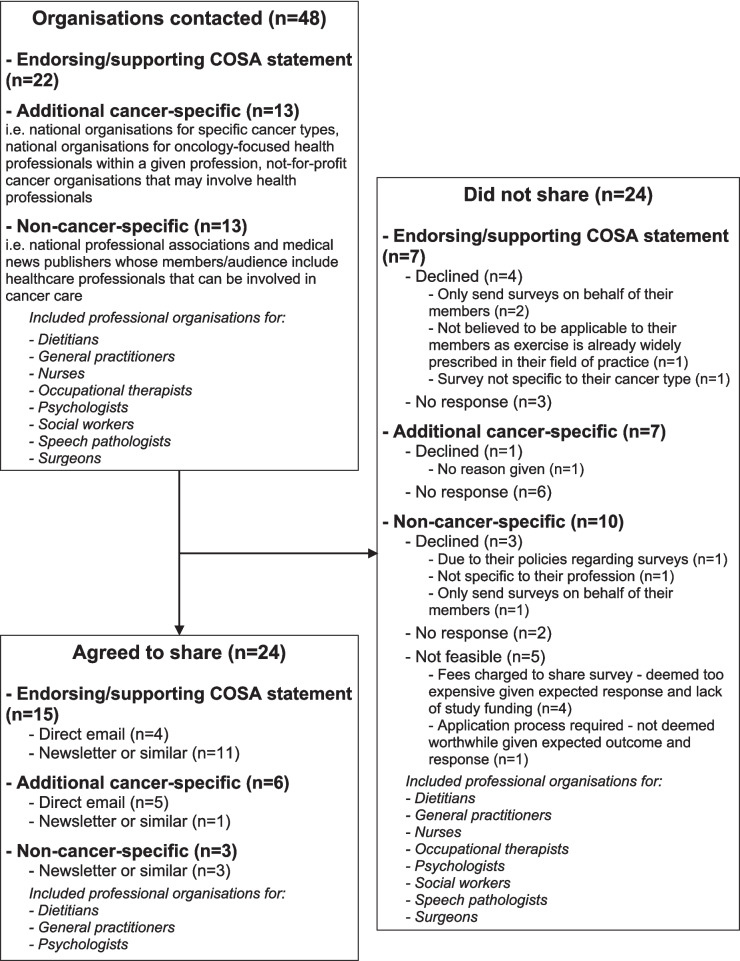
Study recruitment approach, including organisations contacted and reached

Given the COSA guidance is directed at “all healthcare professionals involved in the care of people with cancer” [4], we aimed to reach as many different healthcare professionals as possible. To achieve this, a systematic and rigorous recruitment approach was used (Fig. 1). An initial email was sent to specifically identified healthcare organisations and target groups within Australia to explain the aim of the survey and request that it be shared with their members. Organisations contacted included those that endorsed or supported the COSA position statement, and additional cancer-specific organisations (e.g. national organisations for specific cancer types, national organisations for oncology-focused health professionals within a given profession, not-for-profit cancer organisations that may involve health professionals), as well as non-cancer-specific organisations (e.g. national professional associations, medical news publishers) whose members/audience include healthcare professionals that can be involved in cancer care. Up to two follow-up emails over approximately 6 weeks were sent to groups that did not respond to initial contact. Participation in the survey was voluntary, and no compensation was offered for completion. As healthcare professionals may have been members of multiple target organisations, the survey invitation reminded participants they may have already received the invitation, and not to repeat the survey if so. The survey remained open until all recruitment avenues were exhausted and no new responses were received for approximately 2 weeks.

### Data analysis

#### Quantitative

Survey responses were collected using Qualtrics, then exported to IBM SPSS (Version 27, IBM Corp., Armonk, NY, USA) for analysis. For partially complete responses, data for all completed questions were included. Data were analysed using descriptive statistics, presented as the number and proportion of participants.

#### Qualitative

Key information describing barriers and facilitators to incorporating exercise into clinical care were extracted from the open-text question responses independently by two authors (FC and MAK). The data were inductively coded and categorised into themes using reflexive thematic analysis, following Braun and Clarke’s approach [12, 13]. The themes were reviewed and discussed by all authors to obtain a final consensus. The authors completing the qualitative analysis have an interest in exercise and its implementation in oncology.

## Results

### Reach

Forty-eight organisations were contacted to distribute the survey. Half (*n* = 24) agreed, eight declined, and 11 did not respond (Fig. 1). The remaining five organisations involved fees or application processes that the research team did not consider feasible given the lack of study funding. Most organisations that agreed to share the survey were not-for-profit organisations (*n* = 19, 79%). They also most commonly shared the survey in a newsletter or similar generic group e-correspondence (*n* = 15; 63%). Given the varied recruitment avenues, we are unable to determine the total number of people who saw/received the study invitation. For the nine organisations who shared the survey via email, approximately 1400 people were sent an email, though the read rate was not shared with us. For organisations who shared the survey via newsletter or other e-correspondence, we do not know the number of people who received the newsletters/e-correspondence, or the number of those that actually opened them and/or saw the study advertisement within.

A total of 152 responses were received between February and September 2023. Of these, ten were ineligible based on initial screening questions, and nine did not complete any questions beyond the initial screening questions. This left 133 responses for analysis. Of these, 113 were complete (every question answered) and 20 were partially complete (responses to between 1 and 5 of the 6 survey sections). As we do not know the number of people who saw/received the invitation to complete the survey, we are unable to calculate the response rate.

### Participant characteristics

Most participants were women (74%), most were involved in cancer care for > 10 years (63%), half were working exclusively with cancer patients (50%), most were working in a public hospital setting (69%), and most were working and in a metropolitan area (64%) (Table 1). Common occupations included nurses (35%), oncologists (26%) and dietitians (8%). Almost all (97%) male participants were medical specialists. Most dietitians (90%) and nurses (98%) were female, while less than half (46%) of the oncologists were female. Common cancer types being treated included breast (56%), lung (36%), colon and rectal (32%), and prostate (26%). Only 28% of participants were current COSA members, while another 13% had been previously.

**Table 1:** Participant characteristics

Demographics	*n* (%)
Total responses	133
Gender	
Woman or female	99 (74.4)
Man or male	33 (24.8)
Prefer not to answer	1 (0.8)
Age	
29 years or younger	10 (7.5)
30–39 years	28 (21.1)
40–49 years	40 (30.1)
50–59 years	41 (30.8)
60 years or older	14 (10.5)
Occupation	
Medical specialist	60 (45.1)
Oncologist	35 (26.3)
Medical oncologist	22 (16.5)
Radiation oncologist	11 (8.3)
Nuclear oncologist	1 (0.8)
Paediatric and adolescent oncologist	1 (0.8)
General practitioner	3 (2.3)
Cardiologist	2 (1.5)
Other	20 (15.0)
Pulmonologist/respiratory physician/thoracic medicine	9 (6.8)
Geriatrician	1 (0.8)
Surgeon	5 (3.8)
Palliative medicine	2 (1.5)
Haematologist	2 (1.5)
Pathologist	1 (0.8)
Nurse	47 (35.3)
Dietitian	10 (7.5)
Other	16 (12.0)
Radiation therapist	2 (1.5)
Social worker	5 (3.8)
Pharmacist	3 (2.3)
Psychologist	2 (1.5)
Breast physician	2 (1.5)
Services manager	1 (0.8)
Nuclear medicine technologist	1 (0.8)
Setting*	
Private hospital	36 (27.1)
Public hospital	92 (69.2)
Private cancer centre	17 (12.8)
Private clinic	23 (17.3)
Academic institution	9 (6.8)
Other	19 (14.3)
Rural or regional	
Yes	48 (36.1)
No	85 (63.9)
State or territory	
Australian Capital Territory	3 (2.3)
New South Wales	30 (22.6)
Northern Territory	0 (0.0)
Queensland	27 (20.3)
South Australia	32 (24.1)
Tasmania	1 (0.8)
Victoria	16 (12.0)
Western Australia	24 (18.0)
Years involved in cancer care	
Less than 2 years	8 (6.0)
2–5 years	18 (13.5)
5–10 years	23 (17.3)
More than 10 years	84 (63.2)
Proportion of patients who are cancer patients	
< 25%	13 (9.8)
26–50%	14 (10.5)
51–75%	10 (7.5)
76–99%	30 (22.6)
100%	66 (49.6)
Most common cancer types among patients involved in care of*	
Bladder	5 (3.8)
Breast	75 (56.4)
Colon and rectal	42 (31.6)
Endometrial	4 (3.0)
Kidney	4 (3.0)
Leukaemia (all types)	14 (10.5)
Liver and intrahepatic bile duct	8 (6.0)
Lung (including bronchus)	48 (36.1)
Melanoma	13 (9.8)
Non-Hodgkin lymphoma	9 (6.8)
Pancreas	18 (13.5)
Prostate	35 (26.3)
Thyroid	4 (3.0)
Other	42 (31.6)
COSA member	
Yes	37 (27.8)
No	79 (59.4)
Have been in the past but not currently	17 (12.8)

*Could select multiple responses so totals do not match total survey participants

### Awareness and practice regarding COSA position statement

**Fig. 2:**
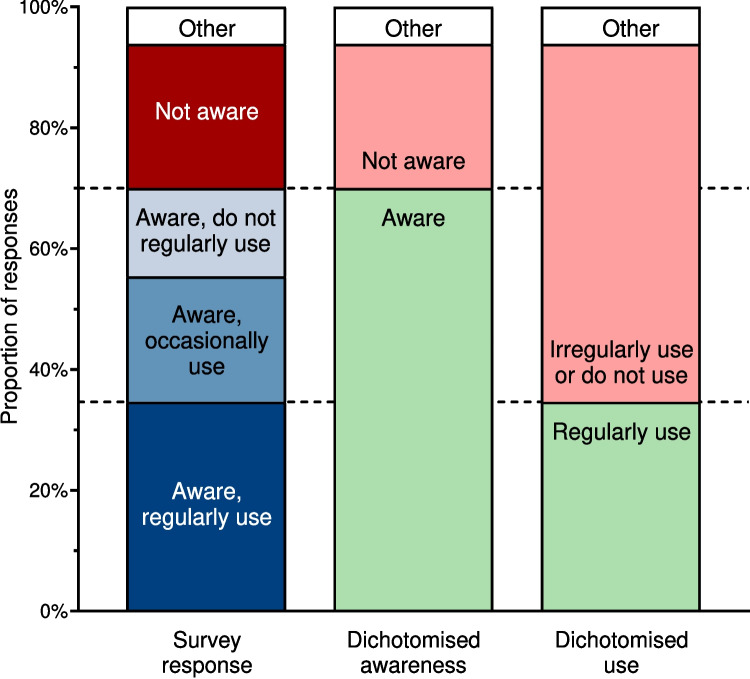
Awareness and use of the COSA position statement (*n* = 130). Dotted horizontal lines indicate how survey responses (column 1) were dichotomised for awareness (column 2) and use (column 3). COSA = Clinical Oncology Society of Australia

**Fig. 3:**
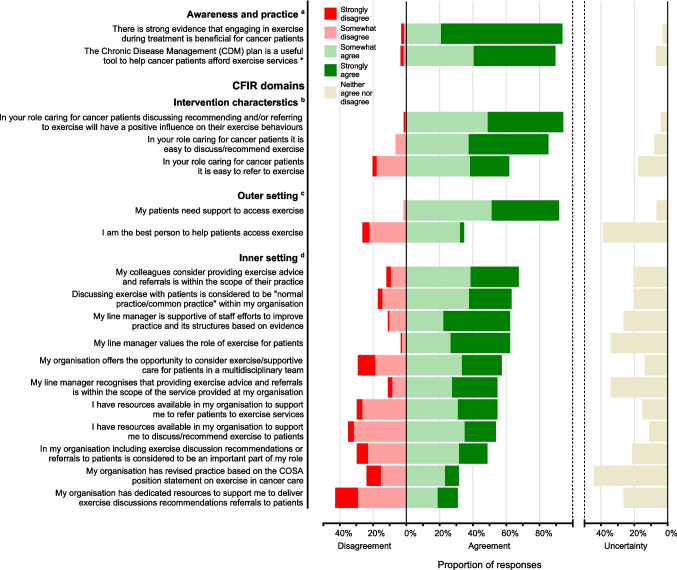
Level of agreement with Likert scale survey items, ranked by overall agreement within each survey section. Each horizontal bar, including *neither agree nor disagree* presented on the right axis, totals 100%. ^a^*n* = 130, ^b^*n* = 123, ^c^*n* = 121, ^d^*n* = 117. *Excludes 16 participants who were not aware the CDM plan was an option for their patients. CDM = Chronic Disease Management, CFIR = Consolidated Framework for Implementation Research, COSA = Clinical Oncology Society of Australia

Responses to all survey questions about exercise in cancer care are presented in the Supplementary Information section. Almost all participants (94%) agreed, either somewhat or strongly, that there is strong evidence that exercise is beneficial for people with cancer. While most participants (70%) were aware of the COSA position statement, fewer (35% overall, 49% of those who were aware) regularly used it in clinical practice (Fig. 2). In Australia, subsidised allied health services (including exercise programs provided by accredited exercise physiologists or physiotherapists) are available to people with chronic disease (including cancer) via Medicare (Australia’s universal health insurance scheme) using a Chronic Disease Management plan. The Chronic Disease Management plan was considered useful to help cancer patients afford exercise services by 79% of participants, while 12% did not know it was an option for their patients (Fig. 3).

### CFIR domains

#### Intervention characteristics

**Fig. 4:**
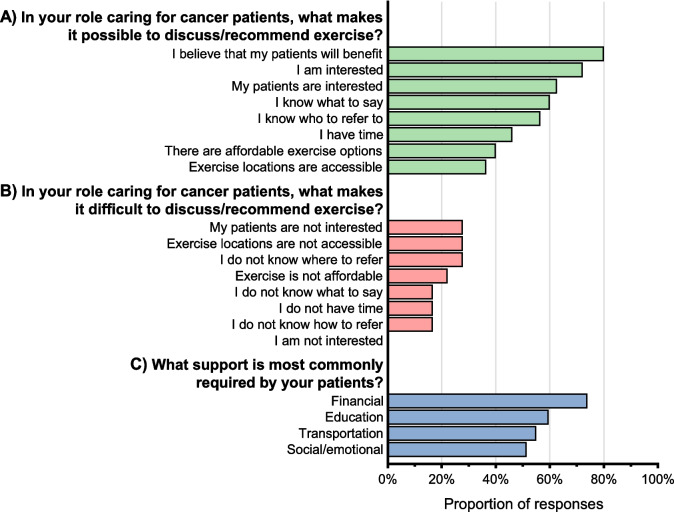
**A** Facilitators and **B** barriers reported for discussing/recommending exercise, and **C** support required by patients. Participants could select multiple responses, so totals do not equal 100%. Responses of *other* are not shown. **A** Asked to 115 participants who strongly agreed, somewhat agreed, or neither agreed nor disagreed that it is easy to discuss/recommend exercise. **B** Asked to 18 participants who strongly disagreed, somewhat disagreed, or neither agreed nor disagreed that it is easy to discuss/recommend exercise. **C** Asked to 111 participants who somewhat agreed or strongly agreed that patients need support to exercise

Most participants (94%) agreed that discussing, recommending, and/or referring patients to exercise will positively influence exercise behaviours. Most (85%) agreed it is easy to discuss or recommend exercise, but fewer (62%) agreed it is easy to refer their patients to exercise. A belief that patients will benefit (80%), an interest in exercise (among both healthcare professionals [72%] and patients [63%]), and knowing what to say (60%) were the most reported factors making discussing/recommending exercise possible (Fig. 4). Conversely, patients not being interested (28%), inaccessible exercise locations (28%), and participants not knowing where to refer patients (28%) were the most common factors reported to make discussing/recommending exercise difficult.

#### Outer setting

Almost all participants (92%) agreed that their patients need support to access exercise; however, few (35%) believed they were the best person to provide this support. The most common types of support participants reported their patients needing were financial (74%), transportation (55%), education (60%), and social/emotional (51%).

#### Inner setting

Most participants (62–68%) agreed their colleagues, manager, and/or organisation values the role of exercise, considers exercise advice and referrals to be within their scope of practice, and is supportive of changing practice based on evidence. However, fewer (49%) agreed that including exercise discussions, recommendations, or referrals to patients is considered an important part of their role. Of note, 76% of nurses and 52% of oncologists agreed that this was an important part of their role, compared to 11% of dietitians and 24% of non-oncologist medical specialists. Over half (57%) agreed their organisation offers the opportunity to consider exercise/supportive care for patients in a multidisciplinary team. Over half (54–55%) agreed they have resources available in their organisation to support them to discuss/recommend exercise and to refer patients to exercise, but fewer (31%) agreed their organisation has dedicated resources available specifically to support this. Only 24% of participants agreed their organisation revised practice based on the COSA position statement.

#### Individual characteristics

Most participants (78%) believed they themselves consistently met national physical activity guidelines.

### Open-ended responses

One-third of participants who reached the final question (*n* = 37; 33%) provided an open-text response. Three key themes were developed relating to barriers to delivering exercise guidance: (1) patient appropriateness, (2) financial challenges, and (3) delivery of care issues (see Supplementary Information section for elaboration on these themes).

## Discussion

This cross-sectional study exploring healthcare professional perspectives on providing exercise in cancer care across four domains of the CFIR framework revealed the highest concentration of barriers within the inner setting of healthcare organisations. Our survey highlights three important issues for consideration: (1) advocacy statements need to be operationalised to create change, (2) understanding implementation challenges for individual contexts is necessary to support integration of exercise into cancer care, and (3) multidisciplinary collaboration is necessary to facilitate exercise pathways.

Advocacy efforts, such as COSA’s position statement on exercise in cancer care, have an important role in changing clinical practice. These statements serve to publicly present the official point of view of influential organisations, which amplifies the view of key stakeholders [9]. However, dedicated work is required to ensure the intent of advocacy statements is actioned. A 2015 review describing barriers of cancer practitioners discussing physical activity with their patients noted that while most practitioners were aware of the benefits and interested in promoting physical activity, yet only a minority did so in practice [14]. Despite the emergence of multiple advocacy statements calling for cancer clinicians to incorporate exercise in standard treatment protocols in the decade since this review was published [4, 15–17], it seems limited progress has been made. Our study demonstrated that while the intent of the COSA position statement (i.e. to promote the integration of exercise as part of standard practice in cancer care) had strong support from participants (94%), only about one third were aware of the statement and regularly actioned it in practice. These findings align with the findings of Shimizu et al. [18] who surveyed 1029 oncology care providers in Japan to understand the impact of the Japanese Breast Cancer Society’s detailed physical activity recommendation and found only about 20% of the providers were aware of, and routinely provided, physical activity information to patients in practice. The low rates of awareness and use of exercise guidance described in both oncology surveys mirror exercise referral rates in healthcare more broadly, as there is a well-recognised evidence-to-practice gap among physicians for routinely recommending physical activity [19–21]. For example, in a survey of nearly 1800 Australian adults, just 18% reported receiving a physical activity recommendation from their general practitioner [22]. While this number increased to 54% among those with poor mental and physical health-related quality of life, this highlights the complexity of integrating exercise referrals into healthcare systems [23] and reinforce the need to improve implementation strategies to facilitate provision of exercise guidance among healthcare practitioners.

Implementing the directives of advocacy statements requires a clear understanding of what barriers are preventing action so that specific strategies can be employed to create change [24]. Researchers have previously identified a myriad of potential barriers to integrating exercise into cancer care across the healthcare system [6]. Barriers commonly noted in our findings mirror those of Shimizu et al. [18], including limited resources to support provision of exercise, lack of awareness about the recommendation, and uncertainty about who is best suited to discuss exercise with patients. Notably, both surveys found participants largely supported providing exercise to patients, which aligns with findings of others [25, 26]. This finding suggests there is recognition among clinicians regarding the benefits of, and need to include exercise in, cancer care. The high level of support for exercise implies implementation efforts need to expand beyond general clinician education to address the specific reasons for low clinician engagement.

It is difficult to gain a clear understanding of the support for and issues related to using the COSA exercise position statement in practice, in part, because of the challenges of engaging clinicians to share their perspectives. It is notable that just half of the organisations contacted for this study agreed to share the survey, and most (*n* = 15; 63%) did so through passive methods (e.g. organisation newsletter) rather than via a direct communication to specifically highlight this as an important opportunity for their members. This low level of engagement coupled with the low response rate (< 10%) of our and other similar surveys investigating exercise awareness and practices among oncology physicians suggests engaging non-exercise focused clinicians in research about exercise in cancer care is difficult [18, 25]. Results of these surveys are likely representative of those most motivated to provide exercise as part of cancer care. Further, despite the consistent low rates of exercise referrals among oncology clinicians [2, 3], one organisation declined to share the survey because “exercise is already widely prescribed in their field of practice”. This lack of awareness by organisational gatekeepers hinders efforts to access views of people who are potentially unsupportive of integrating exercise into care. As efforts for widespread provision of exercise in cancer care continue to develop, it is important to recognise these potential gaps. Generalised insight from large surveys can guide further exploration of issues; however, it is not enough to drive plans for change. Our survey suggests individual organisations are where most barriers are present. Therefore, it is critical to gain an understanding the unique issues impacting the people and places within the organisations targeted for change so specific implementation strategies can be developed.

Successful integration of exercise into cancer care will require support across multiple stakeholder groups. An implementation agenda developed by a group of international exercise oncology experts highlights roles for researchers, consumers, policy makers, clinicians, and exercise oncology professionals in achieving this [7]. Integration of exercise into cancer care will require continued efforts to gain support from all relevant stakeholder groups. While the COSA position statement is currently endorsed by 20 groups [4], continuing to expand the breadth of groups engaged in advocacy efforts may be a useful step toward achieving more widespread implementation. For example, engaging general practitioners will allow the potential of the Chronic Disease Management plan financial support pathway to be realised, which is an important issue given that patient financial barriers highlighted as a challenge by most respondents. Further, implementation strategies should clearly describe who is responsible for what roles [27]. Given most survey participants were unsure whether they were the best person to help patients access exercise, and there was a large discrepancy among healthcare professional disciplines (i.e. most nurses and oncologists considered it a part of their role; few professionals in other roles saw it as their responsibility), it is clear implementation efforts should aim to specify whose responsibility it is to deliver exercise information, acknowledging there is not a one-size-fits-all solution. Multiple people may be appropriate to discuss, recommend, or refer to exercise depending on the context. As research continues toward understanding best practice implementation strategies, it is important to also consider the perspectives of exercise professionals who deliver exercise to people with cancer, as well as people with cancer themselves, neither of which were included in the current study. Co-design strategies that include consumers, organisational leaders, and clinicians should be considered to enhance the feasibility of the strategies [28].

Several limitations should be considered. The survey is cross-sectional so it is unable to determine causality or changes in practice over time. The results are subject to volunteer bias with oncology health professionals with an interest in exercise more likely to participate. Despite being anonymous, the data may also be subject to response bias. Not all stakeholder groups are represented, despite deliberate efforts to recruit widely. Due to the recruitment approach used, the exact response rate is unknown, but it is estimated to be low. The study is specific to Australian healthcare professionals and an Australian position statement, although the findings may be generalisable to other developed countries with similar national statements.

In conclusion, despite high agreement that exercise is beneficial in cancer care and that COSA’s guidance is useful for patients, only a minority of oncology healthcare professionals routinely apply exercise recommendations in practice. Targeted efforts to overcome barriers that impact implementation of guidelines into practice are needed to improve incorporation of COSA’s exercise recommendations to be integrated into standard cancer care.

## Supplementary Information

Below is the link to the electronic supplementary material.

Supplementary file1 (DOCX 36 KB)

## Data Availability

The de-identified data we analysed are not publicly available, but requests to the corresponding author for the data will be considered on a case-by-case basis.
