# Supplementary material for: Translating advocacy into action: exploring oncology healthcare professionals’ awareness and use of the Clinical Oncology Society of Australia position statement on exercise in cancer care
Source: Support Care Cancer. 2025 Jun 14;33(7):581. doi: 10.1007/s00520-025-09633-0 (PMC12167252; doi:10.1007/s00520-025-09633-0)
Supplement: Supplementary file 1 — Supplementary file1 (DOCX 36 KB) [file 520_2025_9633_MOESM1_ESM.docx]

**Supplementary Information**

**RESULTS**

**Open-ended responses**

Three key themes were developed, primarily focused on barriers to delivering exercise guidance.

1. Patient appropriateness: *“The call goes unheeded”*

There was a perception that patients were “*disinterested*” in exercise. Healthcare practitioners cited patients’: 1) dislike of vigorous or structured programs; 2) not seeing exercise as a priority, and 3) lack of time to dedicate to exercise. A concern about patients’ ability to engage in exercise during treatment was also raised:

*“Patients should not feel pressured to exercise when they are on active treatment and*  *feeling very unwell.”*

1. Financial challenges: *“Cost is a deterrent”*

Concern about costs of programming, both at a patient and organizational level, were raised. Direct costs for patients to attend programs without options for financial assistance was noted, particularly in regional areas. The option to access funding through Medicare’s Chronic Disease Management plan was seen as “*problematic*”:

*“There is frustration with having to attend a GP clinic, when they have a clinical*  *nurse consultant or nurse practitioner who has all their relevant documentation and*  *diagnostic information could easily refer them to an EP or physiotherapist.”*

At the organizational level, the lack of money to support the inclusion of exercise into oncology care was raised, with one practitioner noting there is “*no funding from government for any of this implementation.”*

1. Delivery of care issues: *“No in-house expert”*

A lack of organisational resources to support the delivery of exercise during cancer care was noted, with particular emphasis on the absence of an “*in-house expert*” to deliver exercise as doctors are “*too busy to do everything*”. The requirement to refer outside was seen to be difficult for several reasons, including creating an additional financial burden to patients; poorly promoted programs; limited or unsuitable program options (particularly in regional/remote areas); and, most notably, the long waitlists to access exercise professionals and services:

*“The greatest challenge our patients face is accessing structured exercise*  *programmes- either individually or in a group setting- due to long waiting lists.”*

A few facilitators were described and primarily focused on suggested improvements to referral processes, including utilising social workers and GPs to initiate referrals, implementing automatic referrals, and relying on support groups to provide exercise.

**Supplementary Table 2.** Survey responses from all participants to questions about exercise in cancer care.

| **QUESTION** | **RESPONSES** |  |
| --- | --- | --- |
| **AWARENESS AND PRACTICE ^a^** |  | **n (%)** |
| **Which statement best describes your knowledge of the COSA position statement on exercise in cancer care?** | I am not aware of it | 31 (23.8) |
|  | I am aware of it, but do not regularly use it in practice | 19 (14.6) |
|  | I am aware of it, and occasionally use it in practice | 27 (20.8) |
|  | I am aware of it, and regularly use it in practice | 45 (34.6) |
|  | Other | 8 (6.2) |
| **There is strong evidence that engaging in exercise during treatment is beneficial for cancer patients** | Strongly agree | 95 (73.1) |
|  | Somewhat agree | 27 (20.8) |
|  | Neither agree nor disagree | 4 (3.1) |
|  | Somewhat disagree | 2 (1.5) |
|  | Strongly disagree | 2 (1.5) |
| **The Chronic Disease Management (CDM) plan is a useful tool to help cancer patients afford exercise services** | Strongly agree | 56 (43.1) |
|  | Somewhat agree | 46 (35.4) |
|  | Neither agree nor disagree | 8 (6.2) |
|  | Somewhat disagree | 2 (1.5) |
|  | Strongly disagree | 2 (1.5) |
|  | I was not aware that the CDM plan was an option for my patients | 16 (12.3) |
| **CFIR DOMAINS** |  |  |
| **INTERVENTION CHARACTERISTICS ^b^** |  |  |
| **In your role caring for cancer patients, discussing, recommending, and/or referring to exercise will have a positive influence on their exercise behaviours** | Strongly agree | 56 (45.5) |
|  | Somewhat agree | 60 (48.8) |
|  | Neither agree nor disagree | 5 (4.1) |
|  | Somewhat disagree | 1 (0.8) |
|  | Strongly disagree | 1 (0.8) |
| **In your role caring for cancer patients, it is easy to discuss/recommend exercise** | Strongly agree | 59 (48.0) |
|  | Somewhat agree | 46 (37.4) |
|  | Neither agree nor disagree | 10 (8.1) |
|  | Somewhat disagree | 8 (6.5) |
|  | Strongly disagree | 0 (0.0) |
| **In your role caring for cancer patients, what makes it possible to discuss/recommend exercise? *** (if answered strongly agree, somewhat agree, or neither agree nor disagree to previous question) | I know what to say | 69 (60.0) |
|  | I have time | 53 (46.1) |
|  | I am interested | 83 (72.2) |
|  | My patients are interested | 72 (62.6) |
|  | There are affordable exercise options | 46 (40.0) |
|  | Exercise locations are accessible | 42 (36.5) |
|  | I know who to refer to | 65 (56.5) |
|  | I believe that my patients will benefit | 92 (80.0) |
|  | Other | 4 (3.5) |
| **In your role caring for cancer patients, what makes it difficult to discuss/recommend exercise? *** (if answered strongly disagree, somewhat disagree, or neither agree nor disagree to previous question) | I do not know what to say | 3 (16.7) |
|  | I do not have time | 3 (16.7) |
|  | I am not interested | 0 (0.0) |
|  | My patients are not interested | 5 (27.8) |
|  | Exercise is not affordable | 4 (22.2) |
|  | Exercise locations are not accessible | 5 (27.8) |
|  | I do not know where to refer | 5 (27.8) |
|  | I do not know how to refer | 3 (16.7) |
|  | Other | 6 (33.3) |
| **In your role caring for cancer patients, it is easy to refer to exercise** | Strongly agree | 29 (23.6) |
|  | Somewhat agree | 47 (38.2) |
|  | Neither agree nor disagree | 22 (17.9) |
|  | Somewhat disagree | 22 (17.9) |
|  | Strongly disagree | 3 (2.4) |
| **OUTER SETTING ^c^** |  |  |
| **My patients need support to access exercise** | Strongly agree | 49 (40.5) |
|  | Somewhat agree | 62 (51.2) |
|  | Neither agree nor disagree | 8 (6.6) |
|  | Somewhat disagree | 2 (1.7) |
|  | Strongly disagree | 0 (0.0) |
| **What support is most commonly required by your patients? *** (if answered somewhat agree or strongly agree to previous question) | Financial | 82 (73.9) |
|  | Transportation | 61 (55.0) |
|  | Education | 66 (59.5) |
|  | Social/emotional | 57 (51.4) |
|  | Other | 11 (9.9) |
| **I am the best person to help patients access exercise** | Strongly agree | 3 (2.5) |
|  | Somewhat agree | 39 (32.2) |
|  | Neither agree nor disagree | 47 (38.8) |
|  | Somewhat disagree | 27 (22.3) |
|  | Strongly disagree | 5 (4.1) |
| **INNER SETTING ^d^** |  |  |
| **My organisation offers the opportunity to consider exercise/supportive care for patients in a multidisciplinary team** | Strongly agree | 28 (23.9) |
|  | Somewhat agree | 39 (33.3) |
|  | Neither agree nor disagree | 16 (13.7) |
|  | Somewhat disagree | 22 (18.8) |
|  | Strongly disagree | 12 (10.3) |
| **My line manager is supportive of staff efforts to improve practice and its structures based on evidence** | Strongly agree | 47 (40.2) |
|  | Somewhat agree | 26 (22.2) |
|  | Neither agree nor disagree | 31 (26.5) |
|  | Somewhat disagree | 12 (10.3) |
|  | Strongly disagree | 1 (0.9) |
| **My organisation has revised practice based on the COSA position statement on exercise in cancer care** | Strongly agree | 10 (8.5) |
|  | Somewhat agree | 18 (15.4) |
|  | Neither agree nor disagree | 52 (44.4) |
|  | Somewhat disagree | 27 (23.1) |
|  | Strongly disagree | 10 (8.5) |
| **My line manager recognises that providing exercise advice and referrals is within the scope of the service provided at my organisation** | Strongly agree | 32 (27.4) |
|  | Somewhat agree | 32 (27.4) |
|  | Neither agree nor disagree | 40 (34.2) |
|  | Somewhat disagree | 10 (8.5) |
|  | Strongly disagree | 3 (2.6) |
| **My line manager values the role of exercise for patients** | Strongly agree | 42 (35.9) |
|  | Somewhat agree | 31 (26.5) |
|  | Neither agree nor disagree | 40 (34.2) |
|  | Somewhat disagree | 3 (2.6) |
|  | Strongly disagree | 1 (0.9) |
| **My colleagues consider providing exercise advice and referrals is within the scope of their practice** | Strongly agree | 34 (29.1) |
|  | Somewhat agree | 45 (38.5) |
|  | Neither agree nor disagree | 24 (20.5) |
|  | Somewhat disagree | 11 (9.4) |
|  | Strongly disagree | 3 (2.6) |
| **Discussing exercise with patients is considered to be "normal practice/common practice" within my organisation** | Strongly agree | 30 (25.6) |
|  | Somewhat agree | 44 (37.6) |
|  | Neither agree nor disagree | 23 (19.7) |
|  | Somewhat disagree | 17 (14.5) |
|  | Strongly disagree | 3 (2.6) |
| **I have resources available in my organisation to support me to discuss/recommend exercise to patients** | Strongly agree | 22 (18.8) |
|  | Somewhat agree | 41 (35.0) |
|  | Neither agree nor disagree | 13 (11.1) |
|  | Somewhat disagree | 37 (31.6) |
|  | Strongly disagree | 4 (3.4) |
| **I have resources available in my organisation to support me to refer patients to exercise services** | Strongly agree | 28 (23.9) |
|  | Somewhat agree | 36 (30.8) |
|  | Neither agree nor disagree | 18 (15.4) |
|  | Somewhat disagree | 31 (26.5) |
|  | Strongly disagree | 4 (3.4) |
| **In my organisation, including exercise discussion, recommendations, or referrals to patients is considered to be an important part of my role** | Strongly agree | 20 (17.1) |
|  | Somewhat agree | 37 (31.6) |
|  | Neither agree nor disagree | 25 (21.4) |
|  | Somewhat disagree | 27 (23.1) |
|  | Strongly disagree | 8 (6.8) |
| **My organisation has dedicated resources to support me to deliver exercise discussions, recommendations, referrals to patients** | Strongly agree | 14 (12.0) |
|  | Somewhat agree | 22 (18.8) |
|  | Neither agree nor disagree | 31 (26.5) |
|  | Somewhat disagree | 34 (29.1) |
|  | Strongly disagree | 16 (13.7) |
| **CHARACTERISTICS OF INDIVIDUALS ^e^** |  |  |
| **Do you consider yourself physically active?** | Yes | 88 (77.9) |
|  | No | 25 (22.1) |
| * could select multiple responses so totals do not match total survey participants. ^a^ n=130, ^b^ n=123, ^c^ n=121, ^d^ n=117, ^e^ n=113. | | |
